# Accelerometer-Derived Total Activity Counts, Bouted Minutes of Moderate to Vigorous Activity, and Insulin Resistance: NHANES 2003–2006

**DOI:** 10.5888/pcd13.160159

**Published:** 2016-10-20

**Authors:** William R. Boyer, Dana L. Wolff-Hughes, David R. Bassett, James R. Churilla, Eugene C. Fitzhugh

**Affiliations:** Author Affiliations: Dana L. Wolff-Hughes, Division of Cancer Control and Population Sciences, National Cancer Institute, Bethesda, Maryland; David R. Bassett, Eugene C. Fitzhugh, Department of Kinesiology, Recreation and Sport Studies, University of Tennessee, Knoxville, Tennessee; James R. Churilla, Department of Clinical and Applied Movement Sciences, University of North Florida, Jacksonville, Florida.

## Abstract

**Introduction:**

The objective of this study was to compare the associations of accelerometer-derived total activity counts per day and minutes of bouted moderate to vigorous physical activity (MVPA) with insulin resistance.

**Methods:**

The sample included 2,394 adults (aged ≥20 y) from the 2003–2006 National Health and Nutrition Examination Survey. Time spent in MVPA, measured by using 2 cutpoints (≥2,020 counts/min [MVPA_2,020_] and ≥760 counts/min [MVPA_760_]), was calculated for bouts of at least 8 to 10 minutes. Total activity counts per day reflects the total amount of activity across all intensities. Insulin resistance was measured via the homeostatic model assessment of insulin resistance (HOMA-IR) and the quantitative insulin sensitivity check index (QUICKI). Two nested regression models regressed HOMA-IR and QUICKI, respectively, on minutes of bouted MVPA and total activity counts per day. We used an adjusted Wald *F* statistic to illustrate strength of association.

**Results:**

After adjustment for covariates, total activity counts per day was more strongly associated with both HOMA-IR (adjusted Wald *F* = 36.83 , *P* < .001) and QUICKI (adjusted Wald *F* = 29.44, *P* < .001) compared with MVPA_2,020_ (HOMA-IR, adjusted Wald *F* = 4.00, *P* = .06; QUICKI, adjusted Wald *F* = 1.08, *P* = .31).Total activity counts per day was more strongly associated with both HOMA-IR (adjusted Wald *F* = 13.64, *P* < .001) and QUICKI (adjusted Wald *F* = 12.10, *P* < .001) compared with MVPA_760_ (HOMA-IR, adjusted Wald *F* = 1.13, *P* = .30; QUICKI, adjusted Wald *F* = 0.97, *P* = .33).

**Conclusion:**

Our study indicated that total activity counts per day has stronger associations with insulin resistance compared with minutes of bouted MVPA. The most likely explanation is that total activity counts per day captures data on light physical activity and intermittent MVPA, both of which influence insulin resistance.

## Introduction

Insulin resistance is associated with an increased risk for cardiovascular disease (1), diabetes (2), and other metabolic disorders. An estimated 30% of the US adult population is insulin resistant (3). Investigating ways to prevent or delay insulin resistance, researchers have focused on the role of physical activity. Studies have highlighted the importance of both total volume (4) and intensity (5) of physical activity in relation to insulin resistance. One study (5) found a significant difference in insulin response after 7 days of exercise at 70% of maximal oxygen consumption but no difference in those exercising at 50% of maximal oxygen consumption. In contrast, another study (4) found lower levels of insulin resistance independent of intensity among people exercising at 170 minutes per week compared with people exercising 115 minutes per week. Results of cross-sectional studies also indicated that higher levels of moderate to vigorous physical activity (MVPA), both bouted (6) and nonbouted (7), were associated with lower levels of insulin resistance.

Wearable monitors that can assess physical activity on a minute-by-minute basis (8,9) have improved researchers’ ability to examine the relationship between physical activity and various chronic conditions such as insulin resistance (6,7,10). Methods of analyzing accelerometer data allow researchers to classify minutes spent in light physical activity (LPA), moderate physical activity (MPA), and vigorous physical activity (VPA); several cutpoints have been established to measure time spent in these intensities (11). However, recent studies show the limitations of these methods in accurately quantifying time (8,12); for example, these methods miss upwards of 50% of the time spent in MVPA. Because of these limitations, a new metric for quantifying accelerometer-derived data is being used: total activity counts per day (TAC/d). This metric incorporates data on physical activity across all intensities, frequencies, and durations (9).

Only 2 recent studies show that accelerometer-derived measures of TAC/d are associated with insulin resistance (13,14). A study published in 2014 (13) showed that TAC/d was significantly associated with measures of insulin sensitivity derived from the homeostatic model assessment of insulin resistance (HOMA-IR). Furthermore, the use of TAC/d eliminated the association between sedentary time and HOMA-IR. A study published in 2015 of a population-based sample (14) found that TAC/d was more strongly associated with multiple insulin resistance–related risk factors — fasting blood glucose, fasting insulin, and C-peptide — than was bouted MVPA.

Although both bouted and nonbouted MVPA are associated with insulin resistance, the aforementioned studies indicate that TAC/d should be considered when assessing the association between physical activity and insulin resistance. Furthermore, it is necessary measure the total volume of activity, because subsets of total volume, such as MVPA, may not fully illustrate the relationship between physical activity and insulin resistance (9,13). To date, no studies have investigated the associations between objectively measured physical activity and insulin resistance by measuring both MVPA and total volume of physical activity. The objective of this study was to determine which measure — MVPA or TAC/d —is more strongly associated with 2 markers of insulin resistance commonly used in epidemiological research — HOMA-IR and the quantitative insulin sensitivity check index (QUICKI) — in a representative sample of US adults. The results of this study will provide further insight into the relationship of TAC/d and bouted MVPA with insulin resistance and the use of these 2 measures in epidemiologic research.

## Methods

This study used data from the 2003–2006 National Health and Nutrition Examination Survey (NHANES), a cross-sectional survey conducted by the National Center for Health Statistics that uses a complex, multistage sampling design to obtain a representative sample of the US population (15). NHANES was designed to provide national estimates of the health and nutritional status of noninstitutionalized US civilians aged 2 months or older. Our analytic sample (n = 2,394) met the following conditions: 1) they were adult men and women aged 20 years or older; 2) they attended a morning mobile examination center (MEC) after an overnight fast of 8 to 9 hours; 3) if female, they were not pregnant or lactating; 4) they had complete data on all the variables of interest; and 5) they had at least 4 days of accelerometer data with 10 or more hours per day of wear time.

### Accelerometer-derived physical activity

Eligible participants were those who participated in the MEC component of NHANES, were ambulatory, and agreed to participate. Eligible participants were given an ActiGraph model 7164 accelerometer and were instructed to wear the device on the right hip for 7 consecutive days (16). Because the ActiGraph is not waterproof, participants were instructed to remove the device during activities in water, such as bathing or swimming (16). Participants were instructed to remove the device also while sleeping.

Accelerometer data were recorded in 1-minute epochs. Nonwear time was defined as 60 minutes or more of no accelerometer counts, with 2 minutes or fewer of limited movement between zero and 100 counts. Wear time was calculated by subtracting the total nonwear time from 24 hours. Participants who had at least 4 days with 10 hours or more hours of valid wear time per day were included in the analysis (16). Several cutpoints exist to denote minutes of MVPA (11). For our study, we used 2 cutpoints for bouted MVPA. One cutpoint (MVPA_2,020_) was defined as minutes consisting of 2,020 or more activity counts that were accumulated in bouts of at least 10 minutes (with 1–2 minutes allowed below the threshold) (17). A second cutpoint (MVPA_760_) was defined as minutes consisting of 760 or more activity counts that were accumulated in bouts of at least 10 minutes (with 1–2 minutes allowed below the threshold) (18). These cutpoints are consistently applied to NHANES data (11). TAC/d was created by summing up the total activity counts and dividing by the number of valid wear days (14).

### Insulin resistance

Insulin resistance measures were HOMA-IR (19) and QUICKI (20), both of which are used in NHANES data analyses (21,22). The HOMA-IR equation was created as a surrogate marker of insulin resistance based on the negative-feedback loop between the liver and β cells (19). QUICKI is a measure of insulin sensitivity, with lower levels reflecting a greater degree of insulin resistance. The QUICKI method is an equation derived from fasting insulin and glucose levels (similar to HOMA-IR) and is based on the linear relationship of these variables with the gold-standard hyperinsulinemic–euglycemic clamp technique (20). Before calculating HOMA-IR and QUICKI, we corrected for differences between the 2005–2006 NHANES cycle and the 2003–2004 NHANES cycle in measuring insulin and glucose values (23). The assays used to measure insulin and glucose changed between 2003–2004 and 2005–2006. NHANES provides an equation to account for those differences, and it was applied before analyzing the data. Data on HOMA-IR were logarithmically transformed, because they were nonnormally distributed.

### Covariates

We included sociodemographic variables, wear-time variables, and markers of health and behavior as covariates. Sociodemographic variables were age (continuous variable in years), sex (male and female), race/ethnicity (non-Hispanic white, non-Hispanic black, Mexican American, and other), and education (<high school graduate, high school graduate/general educational development [GED], some college, and college graduate). Health and behavior variables included smoking status (current smoker, former smoker [those reporting quitting within the previous 6 months], and nonsmoker), measured waist circumference, hypertension, and diabetes. Waist circumference was treated as a continuous variable. Participants were classified as having hypertension if they had a measured systolic blood pressure of 140 mm Hg or more or a diastolic blood pressure of 90 mm Hg or more (24), self-reported physician-diagnosed hypertension, or were currently taking medication for hypertension. Diabetes status was categorized into 3 levels: no diabetes, prediabetes, and diabetes. Those classified as having diabetes met 1 of the following conditions: answered yes to the question “Has a doctor or health-professional ever told you that you have diabetes?,” were currently taking medication for diabetes, or had a fasting blood glucose 126 mg/dL or more (25). Prediabetes was defined as having physician-diagnosed prediabetes or a fasting glucose ranging from 100 to 125 mg/dL (25). Participants were classified as not having diabetes if they answered no to the question “Has a doctor or health-professional ever told you that you have diabetes?” and having a fasting glucose of less than 100 mg/dL (25).

All analyses were conducted using SAS 9.4 (SAS Institute, Inc). We used the *proc surveyreg* procedure, which accounts for the complex sampling design and survey nonresponse inherent to NHANES (15). Fasting subsample weights were used, thus ensuring the proper subsample for all analyses. Weighted prevalence estimates were generated for all categorical variables. The weighted mean, standard error, and range were generated for all continuous variables.

Age-adjusted linear regressions were performed; these regressed TAC/d and MVPA separately on each measure of insulin resistance. Two nested linear regression models were created. Both physical activity metrics were simultaneously entered into the regression models to examine the difference in the magnitude of the association between each physical activity metric and insulin resistance. Model 1 included all covariates except waist circumference. Model 2 included all covariates from model 1 plus waist circumference. The Wald *F* statistic was used to indicate the strength of association for both physical activity metrics. A larger Wald *F* indicates a stronger association with the dependent variable (26). For the regression models using MVPA_2,020_ and TAC/d, multicollinearity was checked and not found to violate the variance inflation factor threshold (<5) (27,28). In the regression models using MVPA_760_ and TAC/d the variance inflation factor statistic was 5.7, violating the threshold set for the analysis. A variance inflation factor of less than 5 is a conservative statistic, and other studies have used a value of less than 10 (29).

## Results

The mean age of our study sample was 48.3 years; 51.4% were women, 73.5% were non-Hispanic white, 32.1% had some college, 51.4% were nonsmokers, 26.9% had hypertension, and 6.5% had diabetes (Table 1). The age-adjusted linear regressions (Table 2) showed that MVPA_2,020_ was significantly associated with both HOMA-IR (β = −0.01, *P* < .001) and QUICKI (β = 0.0003, *P* < .001). MVPA_760_ was also significantly associated with both HOMA-IR (β = −0.003, *P* < .001) and QUICKI (β = 0.00001, *P* < .001). TAC/d was also significantly associated with both HOMA-IR (β = −0.000002, *P* < .001) and QUICKI (β = 0.00000004, *P* < .001).

**Table 1:** Sociodemographic, Health, and Physical Activity Characteristics of the Study Sample^a^ (n = 2,394), NHANES 2003–2006

Covariate	Value^b^
**Age, mean (SE), y^c^**	48.3 (0.6)
**Sex**	
Male	1,243 (48.6)
Female	1,151 (51.4)
**Race/ethnicity**	
Non-Hispanic white	1,292 (73.5)
Non-Hispanic black	443 (9.7)
Mexican American	485 (7.6)
Other	174 (9.1)
**Education**	
<High school graduate	601 (15.3)
High school graduate/general educational development (GED)	603 (25.8)
Some college	686 (32.1)
College graduate	504 (26.8)
**Smoking status**	
Nonsmoker	1,219 (51.4)
Former smoker (quit within the previous 6 mo)	712 (27.2)
Smoker	463 (21.4)
**Waist circumference, mean (SE), in**	97.9 (0.5)
**Hypertension^d^**	
Yes	818 (26.9)
No	1,576 (73.1)
**Diabetes**	
No diabetes	1,346 (61.3)
Prediabetes^e^	837 (32.2)
**Diabetes^f^**	211 (6.5)
**Physical activity measure^g^**	
Total activity counts per day	268,912 (4,247)
MVPA ≥2,020 counts/min^h^, mean (SE), min	7.0 (0.4)
MVPA ≥760 counts/min^h^, mean (SE), min	15.2 (0.1)
**Insulin resistance**	
Homeostatic model assessment of insulin resistance (HOMA-IR)^i^	2.8 (0.7)
Quantitative insulin sensitivity check index (QUICKI)^j^	0.15 (0.0006)

Abbreviations: MVPA, moderate to vigorous physical activity; NHANES, National Health and Nutrition Examination Survey; SE, standard error.

a The analytic sample (n = 2,394) met the following conditions: 1) they were adult men and women aged 20 years or older; 2) they attended a morning mobile examination center (MEC) after an overnight fast of 8 to 9 hours; 3) if they were female, were not pregnant or lactating; 4) they had complete data on all the variables of interest; and 5) they had at least 4 days of accelerometer data with 10 or more hours per day of wear time.

b All values are number (weighted percentage) unless otherwise indicated.

c Sample size was 2,394.

d Defined as systolic blood pressure ≥140 mm Hg or a diastolic blood pressure ≥90 mm Hg, self-reported physician-diagnosed hypertension, or taking medication.

e Physician-diagnosed prediabetes or a fasting blood glucose range of 100–125 mg/dL.

f Physician-diagnosed, currently taking medication for diabetes, or having a fasting blood glucose ≥126 mg/dL.

g Data for each of these continuous variables were available for all 2,394 participants.

h MVPA was measured in bouted minutes of activity.

i HOMA-IR: (fasting insulin [uU/mL] × fasting glucose [mmol/L])/22.5.

j QUICKI: 1/(log fasting glucose [mg/dL] + log fasting insulin [uU/mL]).

**Table 2:** Age-Adjusted Linear Regression Models: Associations Between Accelerometer-Derived MVPA With Measurements of Insulin Resistance and Total Activity Counts per Day With Measurements of Insulin Resistance, NHANES 2003–2006

Metric	Homeostatic Model Assessment of Insulin Resistance (HOMA-IR)		Quantitative Insulin Sensitivity Check Index (QUICKI)	
	β (SE)	Adjusted Wald *F*	β (SE)	Adjusted Wald *F*
**MVPA ≥2,020 counts/min**	−0.01 (0.002)	61.56^a^	0.0003 (0.004)	43.51^a^
**MVPA ≥760 counts/min**	−0.003 (0.004)	52.89^a^	0.00001 (0.00001)	43.77^a^
**Total activity counts/d**	−0.000002 (0.000000002)	69.84^a^	0.00000004 (0.00000001)	57.38^a^

Abbreviations: MVPA, moderate to vigorous physical activity; NHANES, National Health and Nutrition Examination Survey; SE, standard error.

a *P* < .001.

The nested regression analyses that used both MVPA_2,020_ and TAC/d in the same regression model and adjusted for covariates (excluding waist circumference) showed that TAC/d was more strongly associated with HOMA-IR (adjusted Wald *F* = 58.97, *P* < .001) and QUICKI (adjusted Wald *F* = 46.71, *P* < .001) than was MVPA_2,020_ (HOMA-IR, adjusted Wald *F* = 0.62, *P* = .44; QUICKI, adjusted Wald *F* = 1.10, *P* = .30) (Table 3). After adjustment for waist circumference, TAC/d remained a stronger predictor of HOMA-IR (adjusted Wald *F* = 36.83, *P* < .001) and QUICKI (adjusted Wald *F* = 29.44, *P* < .001) compared with MVPA_2,020_ (HOMA-IR, adjusted Wald *F* = 4.00, *P* = .06; QUICKI, adjusted Wald *F* = 1.08, *P* = .31).

**Table 3:** Multiple Nested Linear Regression: Associations Between Accelerometer-Derived MVPA and Total Activity Counts per Day With Measurements of Insulin Resistance, NHANES 2003–2006

Measurement	Model 1^a^		Model 2^b^	
	β (SE)	Adjusted Wald *F*	β (SE)	Adjusted Wald *F*
**Homeostatic model assessment of insulin resistance (HOMA-IR)**				
MVPA ≥2,020 counts/min	−0.002 (0.001)	0.62	0.002 (0.001)	4.00
Total activity counts/d	−0.000001 (0.0000002)	58.97^c^	−0.000001 (0.0000001)	36.83^c^
**Quantitative insulin sensitivity check index (QUICKI)**				
MVPA ≥2,020 counts/min	0.00004 (0.00004)	1.10	−0.00004 (0.00004)	1.08
Total activity counts/d	0.00000001 (0.00000001)	46.71^c^	0.00000002 (0.00000004)	29.44^c^
**Homeostatic model assessment of insulin resistance (HOMA-IR)**				
MVPA ≥760 counts/min	0.001 (0.001)	2.18	0.001 (0.001)	1.13
Total activity counts/d	−0.000002 (0.0000003)	31.59^c^	−0.000001 (0.0000002)	13.64^c^
**Quantitative insulin sensitivity check index (QUICKI)**				
MVPA ≥760 counts/min	−0.00003 (0.00001)	1.93	−0.00002 (0.00002)	0.97
Total activity counts/d	0.0000001 (0.00000001)	28.00^c^	0.00000003 (0.00000001)	12.10^c^

Abbreviations: MVPA, moderate to vigorous physical activity; NHANES, National Health and Nutrition Examination Survey; SE, standard error.

a Adjusted for age, sex, race/ethnicity, education, smoking status, diabetes, hypertension, wear time, MVPA, and total activity counts per day.

b All model 1 covariates plus measured waist circumference.

c *P* < .001.

The nested regression analyses that used MVPA_760_ and TAC/d and adjusted for covariates (excluding waist circumference) found that TAC/d was more strongly associated with HOMA-IR (adjusted Wald *F* = 31.59, *P* < .001) and QUICKI (adjusted Wald *F* = 28.00, *P* < .001) than was MVPA_760_ (HOMA-IR, adjusted Wald *F* = 2.18, *P* = .15; QUICKI, adjusted Wald *F* = 1.93, *P* = .17) (Table 3). After adjustment for waist circumference, TAC/d remained a stronger predictor of HOMA-IR (adjusted Wald *F* = 13.64, *P* < .001) and QUICKI (adjusted Wald *F* = 12.10, *P* < .001) compared with MVPA_760_ (HOMA-IR, adjusted Wald *F* = 1.13, *P* = .30; QUICKI, adjusted Wald *F* = 0.97, *P* = .33).

## Discussion

The results of this study provide insight into the associations between objectively measured physical activity and insulin resistance. In separate models, both MVPA and TAC/d were significantly associated with insulin resistance. However, the combined models showed that TAC/d was more strongly associated with insulin resistance than was MVPA. Moreover, regardless of the cutpoints used, bouted minutes of MVPA were not significantly associated with insulin resistance in the fully adjusted models.

Our results, which used surrogate measures of insulin resistance, extend the results reported by investigators (14) who found that TAC/d, compared with bouted MVPA, was more strongly associated with insulin resistance–related cardiometabolic risk factors (ie, fasting glucose, fasting insulin, and C-peptide). Using data from the 2003–2006 NHANES, another study (13) found significant associations between TAC/d and HOMA-derived insulin sensitivity or β cell function, independent of inactive time. These results, as well as the results of our study, indicate that TAC/d is important to consider, independent of MVPA, when examining physical activity and insulin resistance.

Two possible explanations exist for why TAC/d is more strongly associated with insulin resistance compared with bouted MVPA. First, TAC/d is not limited by having to classify each minute according to categorical cutpoints that define sedentary time, LPA, MPA, or VPA; instead, activity counts per minute vary along a continuous scale (9,12). Second, TAC/d incorporates the intensity, frequency, and duration of activity, while bouted MVPA represents only a small subset of the overall volume of physical activity. Several studies highlight the independent contribution of LPA (30) and nonbouted MVPA (7,10) in improving insulin resistance profiles. The use of TAC/d accounts for the activity attributable to these other physical activity subcategories, thus strengthening the relationship between physical activity and insulin resistance. TAC/d is more strongly related to cardiometabolic risk than MVPA (14), and our study extends this finding to insulin resistance.

A major strength of this study is the external validity resulting from the use of a large, nationally representative sample. Another strength is the use of validated indices of insulin resistance that are highly correlated with the gold standard euglycemic–hyperinsulinemic clamp technique (19,20). This study also has several limitations. First is the use of bouted MVPA minutes using the 2,020 counts-per-minute cutpoint, which can underestimate time spent in MVPA (12). However, these cutpoints have consistently been used in NHANES analyses (11). Second, although the 760 counts-per-minute cutpoint captures more MVPA, it can also overestimate it (12). Third, physical activity may be underestimated by accelerometry because accelerometers cannot capture data on nonambulatory movements such as swimming and resistance training. Fourth, the counts used in this study were specific to the features of the ActiGraph accelerometer. Thus, these counts cannot be applied to other devices. Fifth, the metric TAC/d has no intuitive meaning (9): although it provides insight into the total volume of physical activity, it does not have the same kind of immediate meaning as the number of minutes, for example. However, population-referenced TAC/d percentiles standardized by age and sex were published in 2015 (31); these percentiles were used to characterize differences in physical activity levels across population subgroups in New York City (32). In the future, criterion-referenced standards for TAC/d may further our understanding of the relationship between physical activity and chronic disease.

In our study of a nationally representative sample of US adults, TAC/d, an index of total activity volume, was more strongly associated with measures of insulin resistance and insulin sensitivity compared with minutes of bouted MVPA using the cutpoints of 2,020 counts per minute and 760 counts per minute. We speculate that the stronger association resulted from the ability of TAC/d to capture counts associated with LPA and nonbouted MVPA, which are both independently associated with insulin resistance (7,10). Future analyses should examine total MVPA defined by the cutpoints used in our study and other common cutpoints to further understand the relationship between components of physical activity and insulin resistance. Finally, our study indicates that it is important to consider physical activity across the entire intensity spectrum when investigating physical activity and insulin resistance.
